# Cobalt metal–organic framework-based ZIF-67 for the trace determination of herbicide molinate by ion mobility spectrometry: investigation of different morphologies†

**DOI:** 10.1039/d0ra09298c

**Published:** 2021-01-12

**Authors:** Mehdi Davoodi, Fatemeh Davar, Mohammad R. Rezayat, Mohammad T. Jafari, Ahmed Esmail Shalan

**Affiliations:** Department of Chemistry, Isfahan University of Technology Isfahan 84156-83111 Iran davar@cc.iut.ac.ir; BCMaterials, Basque Center for Materials, Applications and Nanostructures Martina Casiano, UPV/EHU Science Park, Barrio Sarriena s/n Leioa 48940 Spain a.shalan133@gmail.com ahmed.shalan@bcmaterials.net; Central Metallurgical Research and Development Institute (CMRDI) P.O. Box 87, Helwan Cairo 11421 Egypt

## Abstract

Co-MOF-based zeolitic imidazolate frameworks (ZIF-67) with various morphologies were prepared *via* an innovative way under distinct reaction conditions. By changing the reaction conditions, including the cobalt source, solvent, time, temperature, and linking agent to the cobalt ions, the morphological evolution of Co-MOF-based ZIF-67 was investigated. The Co-MOF-based ZIF-67 was applied as an adsorbent fiber in the solid-phase microextraction (SPME) technique for extracting a herbicide, namely molinate (as a test compound), in aqueous samples. For recognizing the molinate molecules, drift tube ion mobility spectrometry (IMS) was employed as a sensitive, rapid, and simple detection technique. Two essential parameters, namely extraction temperature and extraction time, influenced the extraction efficiency, and these parameters were also analyzed and optimized. The linear dynamic range (LDR) and the determination coefficient were found to be 0.5–20.0 μg L^−1^ and 0.9990, respectively. In this regard, the limit of quantification (LOQ) and the detection limit (LOD) were calculated and found to be 0.5 μg L^−1^ and 0.15 μg L^−1^, respectively. Finally, the effect of the adsorbent with different morphologies on the extraction efficiency was compared.

## Abstract 2

Co-MOF-based zeolitic imidazolate frameworks (ZIF-67) with various morphologies were prepared *via* an innovative way under distinct reaction conditions.

## Graphical abstract

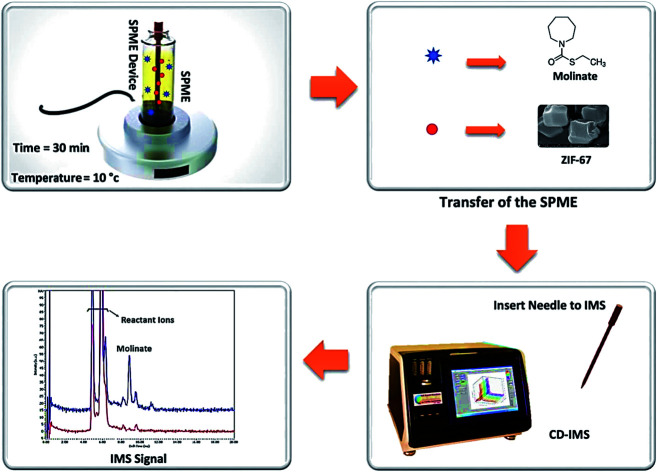

## 1. Introduction

One of the critical worldwide issues that humans struggle with is pesticide contamination.^1^ Quantity and exposure are the two main factors that indicate toxic effects on human health. Pesticides are environmental contaminants that are detected in rivers, surface waters, or soils.^1,2^ Molinate is a thiocarbamate herbicide extensively applied worldwide to protect weeds in rice crops.^3,4^ This herbicide is stable at atmospheric pressure as well as environmental temperature and is a liquid with a pungent odor, and is colorless and transparent. The heat decomposition of this compound produces toxic fumes that contain sulphur, oxygen, and nitrogen.^5^

Furthermore, ion mobility spectrometry (IMS) technique was introduced as a recognizing system.^6^ The IMS pathway depends on the movement of the ions in the constant electric field. The gaseous compounds are ionized and then introduced with an electric shutter into a drift tube. The ionized compounds are moved to the detector in the drift tube depending on the mass, charge, and shape (intrinsic mobility) of the compound. Consequently, the IMS technique could be used for recognizing different blends, including toxic compounds (herbicides, pesticides, and insecticides), warfare agents, and abuse and clinical drugs.^7^

Metal–organic frameworks (MOFs), a class of porous materials, are essential for numerous scientific studies,^8–10^ such as catalysts, gas absorption, drug delivery, separation, electronic devices, and sensors due to their essential features, including large surface area, tunable organic ligands, high crystallization, large pore volume, and adjustable pore size.^11–13^ Zeolitic imidazolate frameworks (ZIFs), as a specific and new category of metal–organic frameworks, consist of metal ions and imidazolate linking agents having intrinsic porosity as well as extraordinary chemical and thermal stability.^14–17^ ZIF materials are obtained *via* a solvothermal method, where ZIFs are prepared from organic solvents.^18,19^ However, organic solvents are often toxic, expensive, flammable, not environment-friendly, and not cost-effective.^19–21^ Accordingly, the production of ZIFs through a green method and low-cost is accordingly desired.^22,23^ The applications of ZIF crystals are highly influenced by the different morphology and size of the as-prepared samples.^23–25^ Numerous parameters such as temperature, duration, concentration, solvent, molar ratio of reactants, and deprotonating agents can affect the morphology and size of the ZIF crystals.^26–28^

In the present study, we have reported various morphologies with a simple pathway for the preparation of Co-MOF-based ZIF-67 nanostructures. Then, to investigate the application of the introduced method, the solid-phase microextraction (SPME) method was applied for extracting the molinate herbicide, as a test compound, in aqueous samples. For recognizing the molinate molecules, ion mobility spectrometry equipped with a corona discharge ionization source was applied. Incidentally, some useful parameters on the extraction efficiency were checked and optimized. Furthermore, we have shown that the use of methanol as a solvent brings the highest extraction efficiency compared to using a mixture of water with methanol or water as a solvent.

## 2. Experimental section

### 2.1. Materials

All chemicals in this project were applied without further purification. CoCl_2_·2H_2_O, Co(SO_4_)·4H_2_O, Co(NO_3_)_2_·6H_2_O, 2-methylimidazole, absolute ethanol, and anhydrous methanol were purchased from Merck company. Herbicide molinate (98% purity) was purchased from Kavosh Kimia Kerman Co., Iran.

### 2.2. Synthesis of Co-MOF-based ZIF-67

Co-MOF-based ZIF-67 samples with various morphologies were synthesized, as stated by the research conducted by a different method.^28^ In brief, 328 mg of 2-methylimidazole (as a linking agent) and 291.05 mg cobalt(ii) nitrate hexahydrate (as a source of Co^2+^) with a stoichiometric ratio of 1 : 4 were dissolved in 20 mL and 10 mL of a mixture of methanol–water and ethanol–water, with the different molar ratio, respectively. Besides, solutions were mixed and stirred at room temperature for 30 min. Then, the resulting purple solution was transferred into a 40 mL Teflon-lined autoclave and heated in a constant temperature drying oven for 30 min at 100 °C. The obtained solution was kept at room temperature for 3 h without stirring. After centrifugation of the resulting solution, the resulting precipitates were collected, washed two times with ethanol and deionized water to remove the potential contaminants, and were dried overnight in an oven at 80 °C. In general, in this study, the effect of distinct reaction parameters, including solvent, reaction temperature, reaction time, cobalt source, and the cobalt to the imidazole molar proportions, was investigated (Table 1).

**Table 1:** Summary of the sample code table of the as-synthesized Co-MOF-based ZIF-67 nanostructures under different reaction parameters

Sample code	Time (h)	Temperature (°C)	Solvent	Cobalt source	2-Methylimidazolate (Hmim)/Co^2+^ molar ratio
HC_1_	12	25	Methanol	CoNO_3_·6H_2_O	1.5/1
HC_2_	12	25	Methanol	CoNO_3_·6H_2_O	2/1
HC_3_	12	25	Methanol	CoNO_3_·6H_2_O	6/1
HC_4_	12	25	Methanol	CoNO_3_·6H_2_O	16/1
SO_1_	12	25	Methanol (M)	CoNO_3_·6H_2_O	4/1
SO_2_	12	25	Ethanol (E)	CoNO_3_·6H_2_O	4/1
SO_3_	12	25	Water (W)	CoNO_3_·6H_2_O	4/1
SO_4_	12	25	W–M (1–2)	CoNO_3_·6H_2_O	4/1
SO_5_	12	25	W–E (1–2)	CoNO_3_·6H_2_O	4/1
CS_1_	12	25	Methanol	CoNO_3_·6H_2_O	4/1
CS_2_	12	25	Methanol	CoCl_2_·2H_2_O	4/1
CS_3_	12	25	Methanol	Co(SO_4_)·4H_2_O	4/1
Te_1_	12	100	W–M (1–2)	CoNO_3_·6H_2_O	4/1
Te_2_	12	100	Water	CoNO_3_·6H_2_O	16/1
Te_3_	12	100	Methanol	CoNO_3_·6H_2_O	16/1
Ti_1_	0.5	25	Methanol	CoNO_3_·6H_2_O	4/1
Ti_2_	6	25	Methanol	CoNO_3_·6H_2_O	4/1
Ti_3_	12	25	Methanol	CoNO_3_·6H_2_O	4/1
Ti_4_	48	25	Methanol	CoNO_3_·6H_2_O	4/1

### 2.3. Characterization of the obtained samples

Fourier transform infrared (FTIR) spectra were obtained with a KBr pellet at a resolution of 4 cm^−1^ using a 680-PLUS FT-IR spectrometer (JASCO, Japan) in the range of 400–4000 cm^−1^. In addition, a JASCO V-750 UV-Spectrophotometer in the range of 400–800 nm was used to investigate the electronic transmissions. Besides, RF-5301pc was applied to measure the photoluminescence spectra of Co-MOFs-based ZIF-67 samples at an excitation wavelength of 490 nm. X-ray diffraction (XRD) patterns were recorded on an X-Pert Pro-MPD Philips with Cu K_α_ radiation (*λ* = 0.15406 nm) at a voltage of 40 kV in the range of 2*θ* = 5–50° with the current of 30 mA. Furthermore, field emission scanning electron microscopy (FE-SEM, FEI model, made in the USA) was used to characterize the morphology of the samples. The BET analysis was described using a BELSORP-mini II apparatus made by MicrotracBEL company (nitrogen adsorption–desorption at 273 k). A Pyris1 device was utilized to evaluate the thermal stability of Co-MOF-based ZIF-67 samples *via* thermometric analysis (TGA) under a nitrogen atmosphere at 10° min^−1^. Corona discharge ionization-ion mobility spectrometry (CD-IMS) as a detection technique was considered and assembled at Teif Azmon Espadana Co, Isfahan, Iran. Furthermore, the instrumental conditions of the CD-IMS are shown in Table S1, in the ESI.†

### 2.4. Preparation of the solid-phase microextraction (SPME) fiber

The wire was immersed in methanol to remove the pollutants from the Ni–Cr wire through the SPME method (SPME fiber). Also, the silicone glue (10% w/v) was prepared in the toluene solvent, and the Ni–Cr wire was placed in this solution for 1 min. Then, the wire was withdrawn from the glue solution and introduced to the ZIF-67 adsorbent for 1 min. Finally, the Ni–Cr wire coated by the ZIF-67 adsorbent was placed at the temperature of 220 °C for 20 mi to remove the toluene solvent and the pollutants.

### 2.5. Evaluation of Co-MOF-based ZIF-67 performance as an adsorbent for the SPME method

**Fig. 1:**
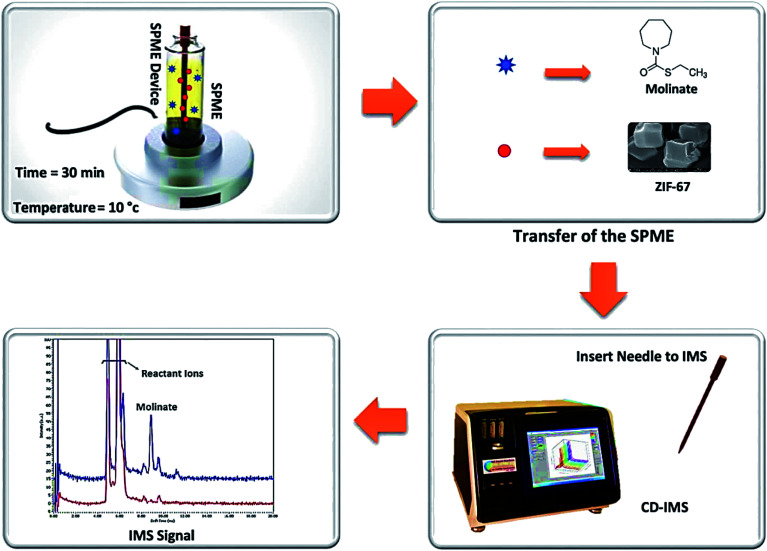
The schematic of the SPME procedure along with the analysis of the molinate by the CD-IMS apparatus.

Fig. 1 shows the schematic of the SPME procedure, along with an analysis of molinate by CD-IMS. Furthermore, 10 mL of the molinate aqueous solution was placed into a 15 mL glass vial as an extraction cell and placed on a stirrer with the temperature set at 10 °C. The proposed adsorbent-coated SPME wire was positioned into the molinate solution for 30 min at a stirring rate of 400 rpm. After achieving the equilibrium time between the adsorbent and sample solution, the ZIF-67 SPME wire was withdrawn and introduced to the CD-IMS injection port (220 °C) at once.

## 3. Results and discussion

**Fig. 2:**
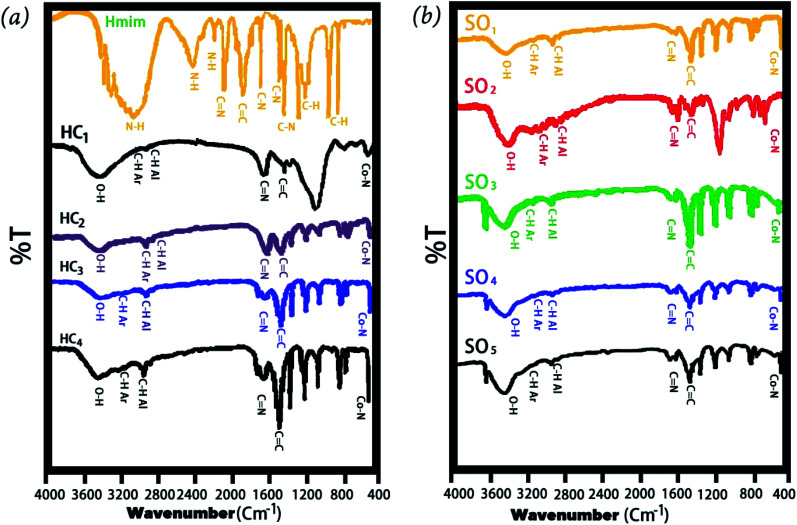
FT-IR spectra of Co-MOF-based ZIF-67, prepared with (a) different Hmim/Co^2+^ molar proportions, Hmim (2-methylimidazolate), HC_1_(1.5), HC_2_(2), HC_3_(6), and HC_4_(16) (b) different solvents, SO_1_ (methanol), SO_2_ (ethanol), SO_3_ (water), SO_4_ (methanol–water), SO_5_ (ethanol–water).

Fig. 2a and b shows the FTIR spectrum of Co-MOF-based ZIF-67 with different solvents and Co-MOF-based ZIF-67 with several Hmim/Co^2+^ molar ratios, respectively. Most of the obtained bands in the Co-MOF-based ZIF-67 are related to the vibration of the imidazole ring and can be interpreted based on the origin of these bands. In the spectrum of Co-MOF-based ZIF-67, the bands at about 3400 cm^−1^ are related to the O–H stretching vibration of the hydroxyl groups.^28,29^ The bands at about 600–1500 cm^−1^ are attributed to the bending and stretching modes of the imidazole ring.^30,31^ Besides, the bands at 2400 and 3000 cm^−1^ in the spectrum of Hmim are assigned to the vibration bending of the N–H functional group.^32^ The bands at about 1570 and 1618 cm^−1^ in the spectrum of Co-MOF-based ZIF-67 and Hmim are attributed to the C=N stretching vibration.^23,33,34^ Besides, the band observed at 1420 cm^−1^ in the spectrum of Co-MOF-based ZIF-67 and Hmim is related to the C=C bond in the imidazole ring.^23,35^ Furthermore, the band observed at about 429 cm^−1^ in the spectrum of Co-MOF-based ZIF-67 is attributed to the Co–N bonds.^36,37^ In the spectrum of Co-MOFs-based ZIF-67, the presence of metal-nitrogen vibrations, absence of the NH bond, and C=N shifted peak to the lower wavenumbers represent considerable effects of the metal ions on the binding agent, confirming the formation of imidazole zeolites (–NH group of Hmim).^36–38^

The UV-Vis spectra of the ZIF-67 prepared using different solvents are given in Fig. S1, in the ESI section.† The interactions between the solvent and the compound cause different maximum wavelengths that affect the electron density on the surface, which is related to the ligand-to-metal charge-transfer (LMCT) characteristics. It demonstrates that the Co-MOFs-based ZIF-67 samples have a blue to red light reaping ability.^34,38^ From Fig. S1, ESI,† we can notice that the lowest absorbance and accordingly the highest transmission was obtained for the sample when water was used as a solvent due to its longer wavelength and lower energy compared to other samples.^38^ The absorbance peaks appeared at 594, 586, 618, and 514 nm for methanol, ethanol, water, and methanol–water, respectively. Furthermore, Fig. S2, ESI† shows the PL spectra of the Co-MOF-based ZIF-67 synthesized with different solvents excited by a laser source at a wavelength of 490 nm.^39^ The PL spectra show emitted light in the range of 540 to 630 nm. Interactions between the solvent and the compound cause different emitted rays in the light range of blue to green. It is observed that when a mixture of ethanol–water is used as a solvent, lower electronic transmission was obtained due to its lower wavelength and more energy compared to other samples.

**Fig. 3:**
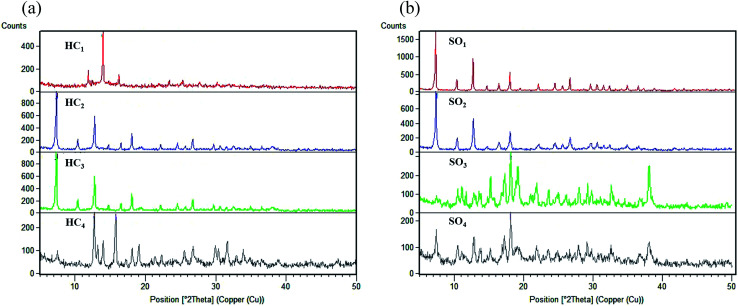
XRD of Co-MOFs based ZIF-67 prepared with (a) different Hmim/Co^2+^ molar ratios, HC_1_(1.5), HC_2_(2), HC_3_(6), and HC_4_(16) (b) different solvents, SO_1_ (methanol), SO_2_ (ethanol), SO_3_ (water), and SO_4_ (methanol–water).

The XRD pattern of Co-MOF-based ZIF-67 prepared by varying the reaction parameters, including the different solvent and different Hmim/Co^2+^ molar proportions, are shown in Fig. 3. The high intensity of the reflections and narrowness as well as the sharpness of these peaks show the high crystallinity percentage of the prepared Co-MOF-based ZIF-67. On the one hand, it is observed that the different Hmim/Co^2+^ molar proportions of the organic ligand to metal ions, HC_1_(1.5), HC_2_(2), HC_3_(6), and HC_4_(16), play an important role in the formation of Co-MOF-based ZIF-67 crystal structures (Fig. 3a). When the proportion of the ligand to the metal ions is low (around 1.5), the as-prepared sample is found to be [Co(OH)_2_] with a layered structure.^40^ By reaching the appropriate molar proportion, pure phase crystals of Co-MOF-based ZIF-67 are formed. On the other hand, for the different solvent SO_1_ (methanol), SO_2_ (ethanol), SO_3_ (water), SO_4_ (methanol–water), and SO_5_ (ethanol–water), we can notice that the XRD pattern of SO_1_ and SO_2_ samples display a different crystal structure as compared to that of other samples. SO_3_ and SO_4_ samples have almost similar XRD patterns matching with prior standard records of Co-MOF-based ZIF-67 materials with a sodalite topology (Fig. 3b).^34,41^

**Fig. 4:**
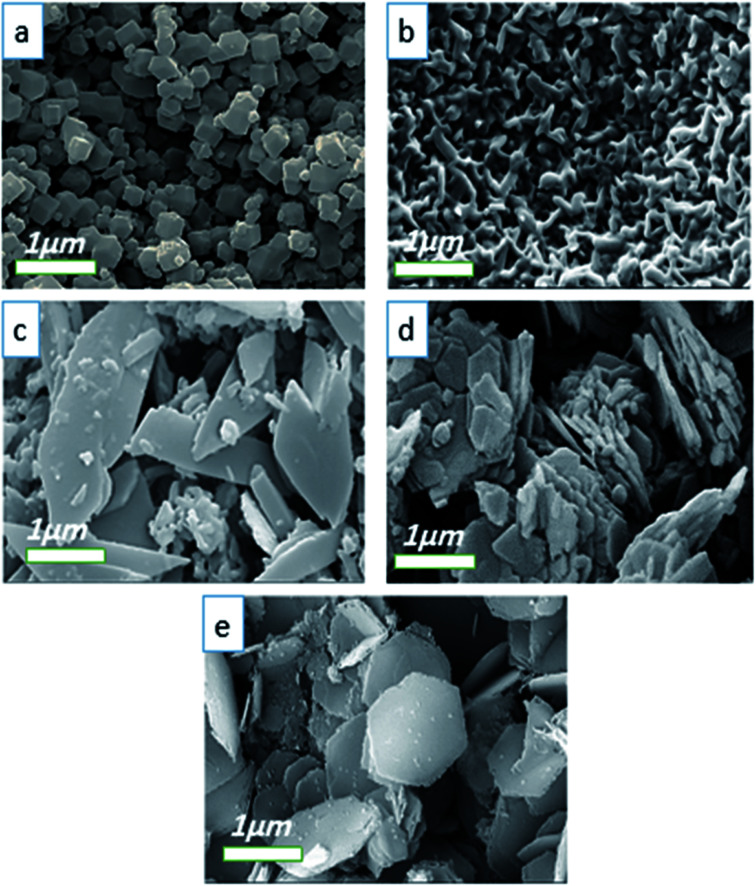
FE-SEM images of Co-MOF-based ZIF-67 prepared with different solvents (a) methanol, SO_1_ (b) ethanol, SO_2_ (c) water, SO_3_ (d) methanol–water, SO_1_, and (e) ethanol–water, SO_5_.

Fig. 4 displays the FE-SEM images of Co-MOF-based ZIF-67 prepared using different solvents: SO_1_ (methanol), SO_2_ (ethanol), SO_3_ (water), SO_4_ (methanol–water), and SO_5_ (ethanol–water). Besides, the particle size distribution of Co-MOF-based ZIF-67 prepared using different solvents, including SO_1_ (methanol) and SO_2_ (ethanol) as examples, is given in Fig. S3, ESI.† Morphology variation is observed by changing the ratio and the type of solvent.^34^ When methanol is applied as a solvent (SO_1_), relatively uniform particles and rhombic dodecahedron morphology with an average size of 344 nm are observed, as shown in Fig. 4a. When ethanol is used as a solvent (SO_2_), the strand-like morphology with a size of 249 nm is observed (Fig. 4b). By using water as a solvent (SO_3_), the leaf-like morphology in two dimensions with larger dimensions than the previous two cases, and a smooth surface is observed (Fig. 4c). In the case of a mixture of water and methanol, the agglomerated hexagonal morphology and pita-like shape are observed (Fig. 4d). The morphology of samples with water and ethanol as solvents (SO_5_) is observed to be similar to the previous one (SO_4_) with a smaller size, as shown in Fig. 4e. Besides, the particle size distribution was calculated by studying the statistical analysis of the as-prepared materials with different types of solvents *via* the free software ImageJ.^42^ The statistical analysis of the particles for Co-MOF-based ZIF-67 obtained with ethanol and methanol as solvents revealed an average size distribution of 250 and 350 nm, respectively, as illustrated in Fig. S3a and b, ESI.† By using a mixture of methanol and water as a solvent, the particles tend to agglomerate, which can be due to the fact that there is less nucleation and more growth, and the resulting particles have a large size. When a mixture of ethanol and water is used, the resulting particles have a distinct hexagonal sheet morphology with a thickness of less than 100 nm.

**Fig. 5:**
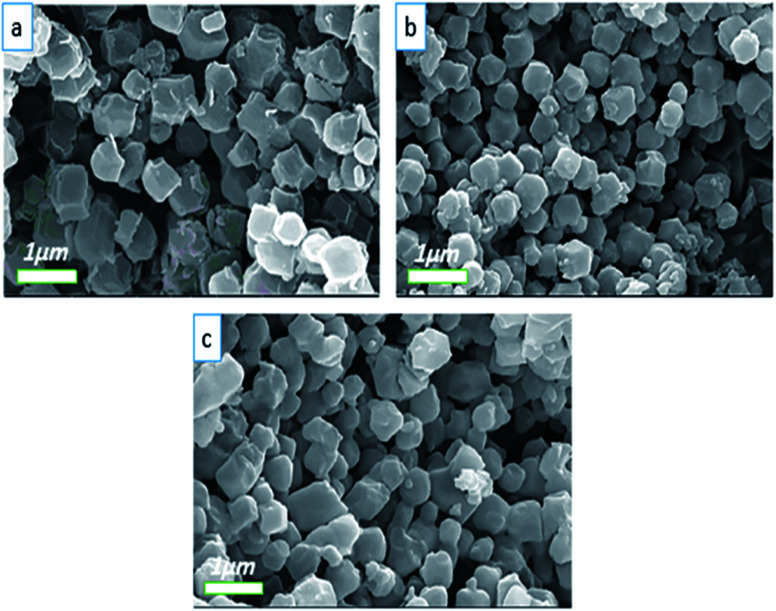
FE-SEM images of Co-MOF-based ZIF-67 prepared with different cobalt sources: (a) Co(NO_3_)_2_, CS_1_, (b) CoCl_2_, CS_2_, and (c) CoSO_4_, CS_3_.

On the other hand, by replacing the cobalt source from Co(NO_3_)_2_ to CoSO_4_ and Co(Cl)_2_, the coordination reactions between cobalt and imidazole probably changed. Due to the different interaction pathways between Co^2+^ and Cl^−^, SO_4_^2−^ and NO_3_^−^, the nucleation rate can be altered.^43^ As a result, when the rate of nucleation changed, we found that particles of different sizes were produced. Another factor is the amount of hydrolysis between cobalt and imidazole, which affects the size and morphology of different crystals of the produced particles. Fig. 5a–c demonstrates the FE-SEM images of Co-MOF-based ZIF-67 prepared with different cobalt sources: Co(NO_3_)_2_, CoCl_2_, and CoSO_4_. The obtained images of the different cobalt source samples indicate the homogeneity of the structure with a cubic like shape. Both the size as well as the morphology of the crystal structures are changed by changing the cobalt source. Furthermore, the average size of ZIF-67 nanostructures prepared with Co(Cl)_2_ as well as CoSO_4_ as cobalt sources, reduced from 404 nm to 287 nm (see Fig. S4, ESI†).

**Fig. 6:**
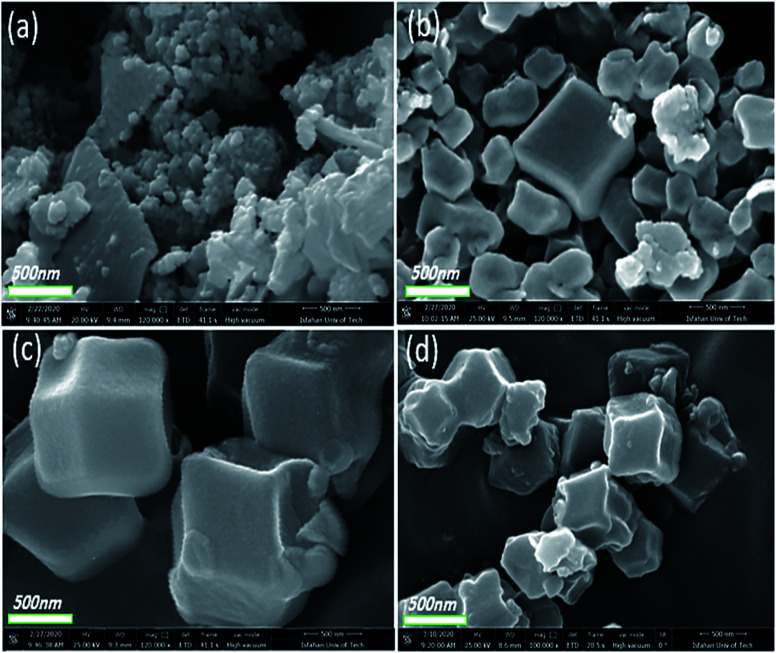
FE-SEM images of Co-MOF-based ZIF-67 prepared with different Hmim/Co^2+^ molar proportions (a) 1.5, HC_1_, (b) 2, HC_2_, (c) 6, HC_3_, and (d) 16, HC_4_.

In general, another factor that can influence the shape of ZIFs is the change in the molar proportion of the ligand to the metal ions.^44,45^Fig. 6a–d displays the FE-SEM images of Co-MOF-based ZIF-67 obtained with different Hmim/Co^2+^ molar proportions of 1.5 for HC_1_, 2 for HC_2_, 6 for HC_3_, and 16 for HC_4_, respectively. Besides, by increasing this proportion, the organic ligands further restrict the bonding between metal ions and prevent their growth. When the molar proportion of imidazole to cobalt is 1.5 to 1, Co-MOF-based ZIF-67 agglomerated particles are formed. Nonetheless, with other samples, a mixture of pseudo-cubic and star-like shapes are formed.

**Fig. 7:**
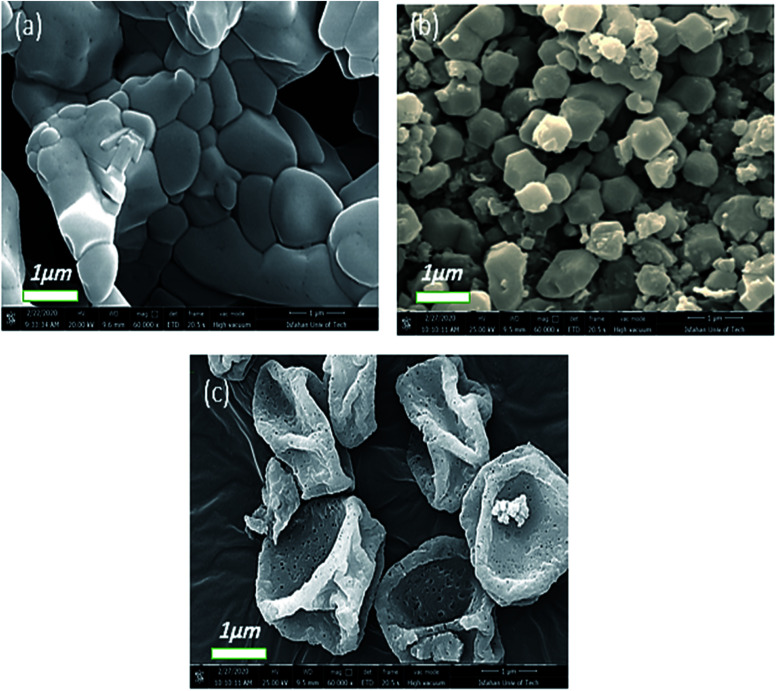
FE-SEM images of Co-MOF-based ZIF-67 prepared with different reaction times (a) 30 min, Ti_1_, (b) 6 h, Ti_2_, and (c) 48 h, Ti_4_.

Consequently, the reaction time is considered another important factor that affects the preparation and morphology of Co-MOF-based ZIF-67, as shown in Fig. 7. According to the SEM images in Fig. 7, when the reaction time increases, the particle size increases, which can be attributed to the Ostwald growth behaviour.^46^ When the reaction time is 30 min (Fig. 7a), Co-MOF-based ZIF-67 particles are agglomerated, but in 6 h (Ti_2_ sample), separated hexagonal shape particles with a diameter of ∼1 μm were obtained (Fig. 7b). Upon increasing the reaction time to 48 h (Fig. 7c), sponge-like hollow, pseudo-spheres were achieved.

**Fig. 8:**
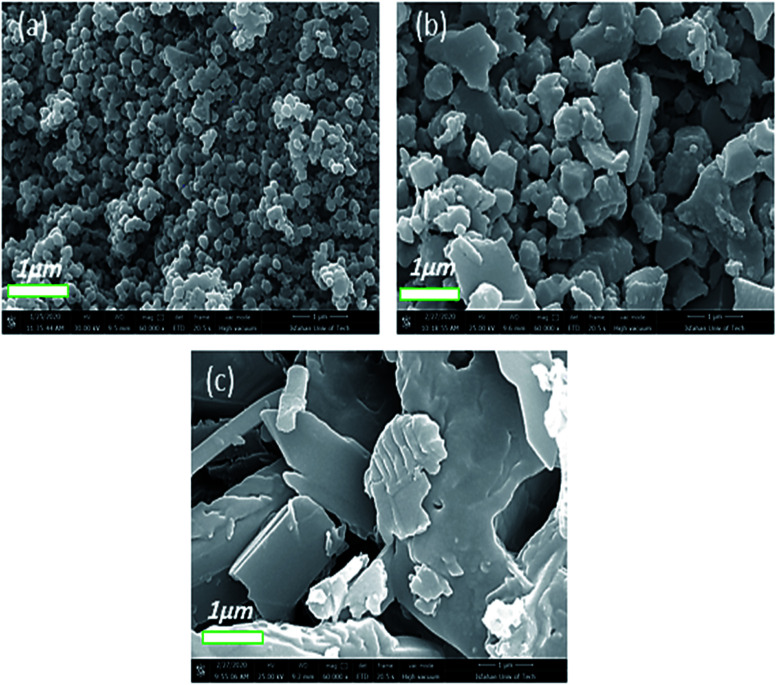
FE-SEM images of Co-MOF-based ZIF-67 prepared at different temperatures indicated as (a) Te_1_, (b) Te_2_, and (c) Te_3_.

Another factor that influenced the properties as well as the morphology of the as-prepared Co-MOF-based ZIF-67 crystals is the reaction temperature. Fig. 8 displays the FE-SEM images of Co-MOF-based ZIF-67 prepared at different reaction temperatures. Former studies confirm the phenomenon, which indicates that the size of the Co-MOF-based ZIF-67 crystals under non-temperature conditions (room temperature) is larger than the crystals under different temperature conditions.^41^ In this study, it is concluded that the reason beyond that phenomenon is the reactants, which are completely dissolved under thermal conditions; therefore, a large number of nuclei were formed and lead to small crystals. Alternatively, nuclei grow relatively slowly at room temperature, leading to large crystals. Therefore, under temperature conditions, the size and morphology of the Co-MOF-based ZIF-67 crystals tend to be more uniform and regular in structure. Besides, the average size of the Co-MOF-based ZIF-67 nanostructures prepared at room temperature (25 °C) was increased from 146 nm to 316 nm rather than those synthesized under the temperature condition of 100 °C (see Fig. S5, ESI†).

**Fig. 9:**
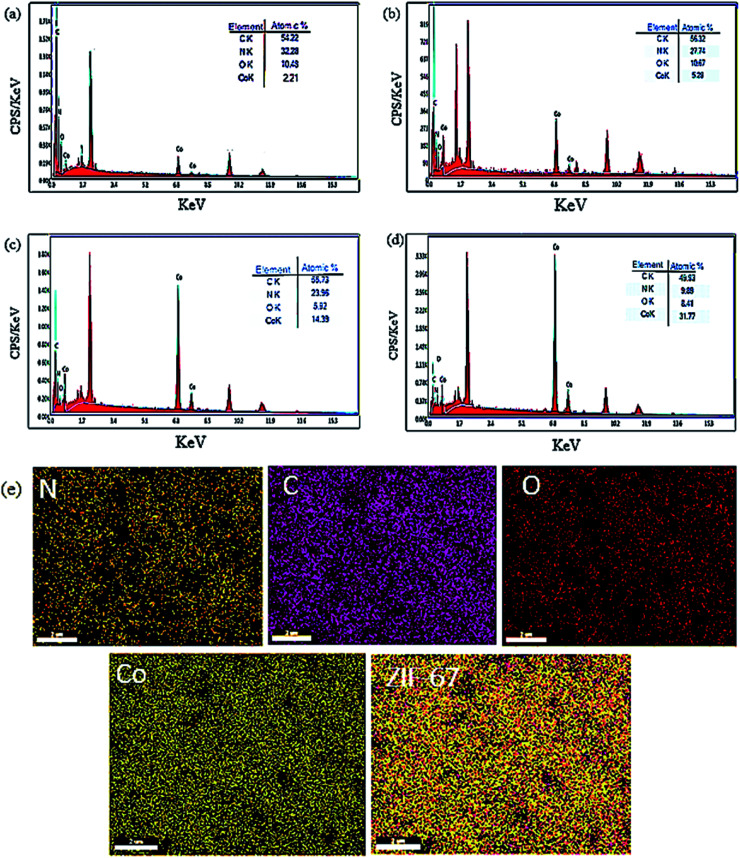
EDX analysis of Co-MOF-based ZIF-67 prepared with several Hmim/Co^2+^ molar proportions (a) 40, (b) 16, (c) 6, (d) 2, (e) X-ray mapping of Co-MOFs-based ZIF-67 for HC_2_.

To gain more information about the composition of the as-prepared materials, the EDX and EDS mapping of the Co-MOF-based ZIF-67 undergoing several Hmim/Co^2+^ molar proportions (Fig. 9) were obtained. It is known that the EDX analysis has an error in detecting elements with a low atomic number.^41^ However, overall, this method confirmed the presence of Co and O, N, C with the desired Hmim/Co^2+^ molar ratios in the as-prepared sample (Fig. 9a–d). The presence of existing elements as well as their uniform distribution in the HC_2_ sample has been confirmed using an X-ray mapping analysis (Fig. 9e). The information gained from the X-ray mapping analysis can affirm the microstructure and properties of the as-prepared materials through the images of the elemental distribution in a sample. Through this technique, we can know the distribution of a particular element without requiring the quantitative point analysis.

**Fig. 10:**
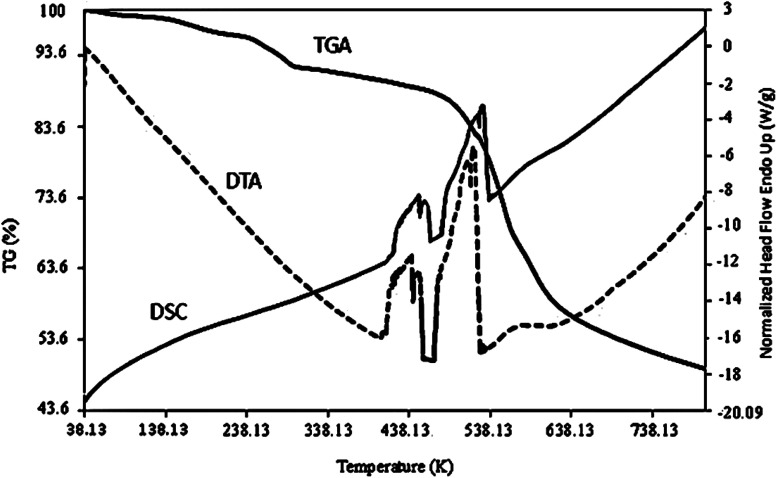
TGA, DSC, and DTA curves of Co-MOF-based ZIF-67 (SO_1_ sample).

Further characterization techniques, including the TGA, DTA, and DSC curves, were performed on the as-prepared Co-MOF-based ZIF-67 (SO_1_ sample) (Fig. 10). It can be seen that the weight loss percent decrease to 200 °C is relevant to the removal of guest molecules and gas molecules (unreacted species). Weight loss in the range of 250–500 °C can be relevant to the decomposition of imidazole ligands. This weight loss emerged as an exothermic peak in the range from 500 to 700 °C in the DTA curve.^30^ In the SPME method, since the adsorbent is exposed to the heat treatment, it is important that its structure is not decomposed at the intended temperature (220 °C). In general, the thermogravimetric curves confirmed that the Co-MOF-based ZIF-67 has good thermal stability during the extraction operations (220 °C).

The BET analysis was used to determine the specific surface area (BET isotherm), size, and empty volume of cavities (BJH), as shown in Fig. S6 and Table S2, ESI.† The as-synthesized sample tendency followed type I isotherm (Langmuir) as per the IUPAC classification.^36^ The pore size distribution for Co-MOF-based ZIF-67 is mainly in the micropore range. The BET surface area for Co-MOF-based ZIF-67 (SO_1_ sample) was 1528 m^2^ g^−1^, along with a pore volume of 0.083 cm^3^ g^−1^ and a mean pore diameter of 1.21 nm.

## 4. Applications of Co-MOF-based ZIF-67

### 4.1. Optimization of the parameters affected by the SPME method

For enhancing the pre-concentration and extraction efficiency, extraction temperature and extraction time as the effective parameters on the proposed method were investigated and optimized.

#### 4.1.1. Extraction temperature and time

**Fig. 11:**
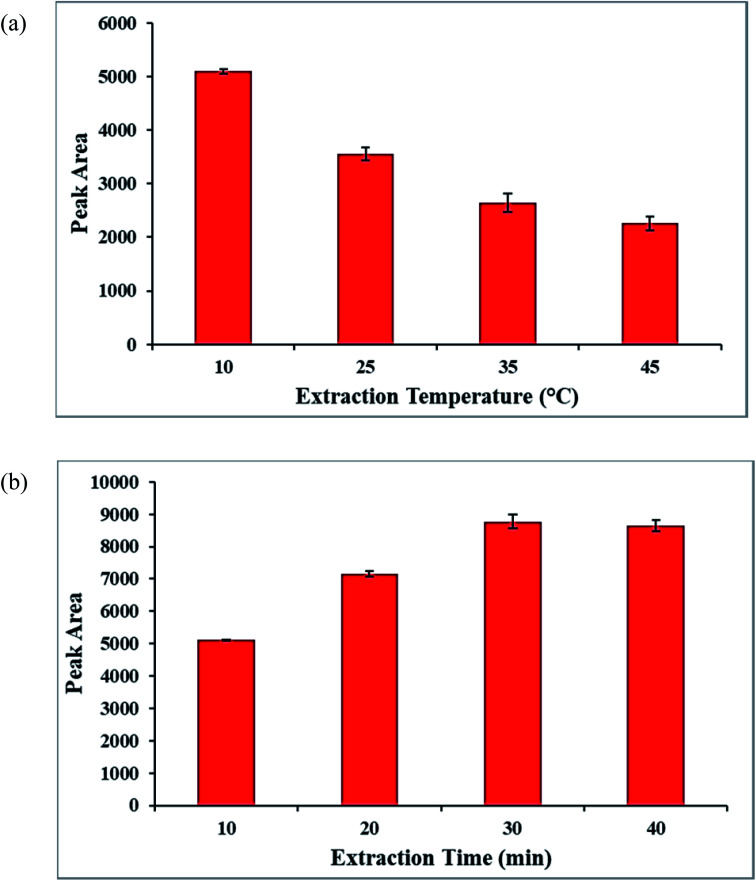
(a) The effect of the extraction temperature (sample solution volume, 10 mL; analyte concentration, 10.0 μg L^−1^), (b) the effect of the extraction time on the extraction efficiency (sample solution volume, 10 mL; analyte concentration, 10.0 μg L^−1^).

Increasing the extraction temperature has an inverse effect for extracting the analyte molecules in the SPME method due to the exothermic process for the adsorption of the analyte molecules on the coated SPME wire.^47^ In this regard, extraction temperature as a pivotal role for the preconcentration of the analyte was studied. To investigate the introduced parameter, the tests were done at the extraction temperatures between 10 °C and 45 °C. According to the acquired data, indicated in Fig. 11a, the temperature of 10 °C was chosen as the best optimized temperature for the next tests.

Besides, to obtain the equilibrium situation between the molinate solution and the Co-MOF-based ZIF-67 SPME wire in the SPME method,^48^ the time of the analyte extraction between 10 and 40 min was investigated and optimized. According to Fig. 11b, the high extraction efficiency for extracting the molinate molecules was obtained at 30 min.

#### 4.1.2. Comparison of the extraction efficiency of the Co-MOF-based ZIF-67 prepared using different solvents

**Fig. 12:**
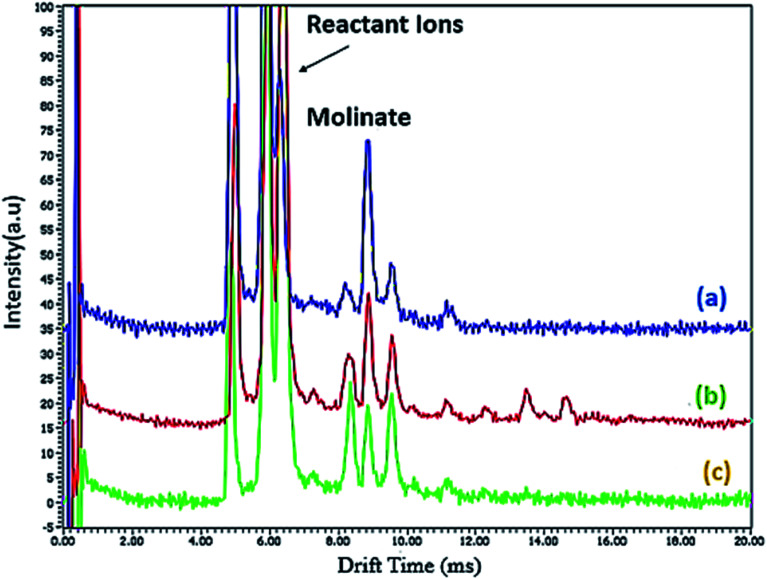
Ion mobility spectra of extracted molinate molecules by different solvents (a) methanol, (b) methanol–water, and (c) water.

To compare the extraction efficiency of the Co-MOF-based ZIF-67 synthesized using different solvents, including methanol, water, and methanol–water mixture, the ion mobility spectrometry test was investigated. Using water as a solvent, the extraction efficiency is low, which is attributed to the leaf-like morphology with a 2D multilayer structure. However, using a mixture of water and ethanol as a solvent for the synthesis of Co-MOF-based ZIF-67, the extraction efficiency is improved. However, because of the agglomerated structure, the extraction efficiency of molinate molecules does not increase optimally. Using methanol as a solvent, a remarkable increase in the extraction efficiency of molinate is observed, which is attributed to the rhombic dodecahedral morphology with a three-dimensional porous structure, micro and meso cavities (Fig. 12). Based on the molinate structure and functional groups in the MOF surface, some interactions are present to adsorb the molinate, such as salt bridge interactions, including the hydrogen bonds and electrostatic interactions between the adsorbent and analyte. Furthermore, the linear dynamic range (LDR) and the determination coefficient were detected to be 0.5–20.0 μg L^−1^ and 0.9990, respectively. In this regard, the limit of quantification (LOQ) and the limit of detection (LOD) were calculated as 0.5 μg L^−1^ and 0.15 μg L^−1^, respectively.

## 5. Conclusions

In summary, a collection of the Co-MOF-based ZIF-67 nanostructures with various morphologies are prepared by a simple method and under different reaction parameters, including reaction solvents, temperature, time, cobalt source, and different Hmim to cobalt ion molar ratios. In this study, Co-MOF-based ZIF-67 has been used as an adsorbent fiber in the solid phase microextraction method to determine the molinate concentration (as a test compound) in the aqueous samples. In this method, an ion mobility spectrometer is used as the detection system. Sensitivity, simple apparatus, and quick analysis are counted as the advantages of this system. Also, two critical parameters, extraction time and temperature, were analyzed and optimized. This study demonstrates that methanol as a solvent brings the highest extraction efficiency compared to water or a mixture of water with methanol as a solvent. Using these optimized conditions, an LOD of 0.15 μg L^−1^, LOQ of 0.5 μg L^−1^, determination coefficient of 0.9990, and LDR of 0.5–20.0 μg L^−1^ were accomplished. The relative standard deviation (RSD) of the different fibers at the molinate concentration of 10 μg L^−1^ was obtained at 6%.

## Author contributions

M. D. helped in preparing the material, characterization by different characterization techniques, and writing the manuscript. M. R. R. helped in the discussion and analytical characterization. M. T. J contributed to the characterization of the obtained materials and discussed the results. Furthermore, F. D. and A. E. S. designed the research, contributed to supervising the work, discussed the results, and wrote the manuscript. All the authors participated in writing, editing, and revising the manuscript.

## Conflicts of interest

The authors declare no conflict of interest.

## Supplementary Material

RA-011-D0RA09298C-s001
