# Expression of concern: Cobalt metal–organic framework-based ZIF-67 for the trace determination of herbicide molinate by ion mobility spectrometry: investigation of different morphologies

**DOI:** 10.1039/d3ra90112b

**Published:** 2023-11-09

**Authors:** Mehdi Davoodi, Fatemeh Davar, Mohammad R. Rezayat, Mohammad T. Jafari, Ahmed Esmail Shalan

**Affiliations:** a Department of Chemistry, Isfahan University of Technology Isfahan 84156-83111 Iran davar@cc.iut.ac.ir; b BCMaterials, Basque Center for Materials, Applications and Nanostructures, Martina Casiano, UPV/EHU Science Park Barrio Sarriena s/n Leioa 48940 Spain a.shalan133@gmail.com ahmed.shalan@bcmaterials.net; c Central Metallurgical Research and Development Institute (CMRDI) P.O. Box 87, Helwan Cairo 11421 Egypt

## Abstract

Expression of concern for ‘Cobalt metal–organic framework-based ZIF-67 for the trace determination of herbicide molinate by ion mobility spectrometry: investigation of different morphologies’ by Mehdi Davoodi *et al.*, *RSC Adv.*, 2021, **11**, 2643–2655, DOI: https://doi.org/10.1039/D0RA09298C.

The Royal Society of Chemistry is publishing this expression of concern in order to alert readers that concerns have been raised regarding the reliability of the XRD data in Fig. 3a and the PL spectroscopy data in Fig. S2. An investigation is underway, and an expression of concern will continue to be associated with the article until a final outcome is reached.

Laura Fisher

2nd November 2023

Executive Editor, *RSC Advances*

## Supplementary Material

