# Supplementary material for: Cobalt metal–organic framework-based ZIF-67 for the trace determination of herbicide molinate by ion mobility spectrometry: investigation of different morphologies
Source: RSC Adv. 2021 Jan 12;11(5):2643–55. doi: 10.1039/d0ra09298c (PMC8693792; doi:10.1039/d0ra09298c)
Supplement: RA-011-D0RA09298C-s001 [file RA-011-D0RA09298C-s001.pdf]

Electronic Supplementary Material (ESI) for RSC Advances.
This journal is © The Royal Society of Chemistry 2020

Supporting Information

Cobalt metal-organic framework based ZIF-67 for trace determination of herbicide molinate by Ion mobility spectrometry: Investigation of different morphologies

Mehdi Davoodi[a], Fatemeh Davar[a,], Mohammad R. Rezayat[a], Mohammad T. Jafari[a] and Ahmed Esmail Shalan[b, c,*]*

[a] Department of Chemistry, Isfahan University of Technology, Isfahan, 84156-83111, Iran

[b] BCMaterials, Basque Center for Materials, Applications and Nanostructures, Martina Casiano, UPV/EHU Science Park, Barrio Sarriena s/n, Leioa 48940, Spain.

[c] Central Metallurgical Research and Development Institute (CMRDI), P.O. Box 87, Helwan, Cairo 11421, Egypt.

**Corresponding authors. E-mail addresses: davar@cc.iut.ac.ir (F. Davar), a.shalan133@gmail.com; ahmed.shalan@bcmaterials.net (A. E. Shalan).*

Table S1: The instrumental conditions of the CD-IMS

Parameter	Setting
IMS mode	Positive
IMS type	Drift tube
Ionization source	Corona discharge
IMS detector	Faraday cup
Needle voltage	2.0 kV
Target electrode voltage	7.0 kV
Drift electric field	400 V cm^{-1}
Drift gas flow (N_2, 99.999 %)	1000 mL min^{-1}
Carrier gas flow (N_2, 99.999 %)	800 mL min^{-1}
IMS cell temperature	150 ºC
Injection port temperature	220 ºC
Drift tube length	11 cm
Shutter grid pulse	180 µs
Shutter grid voltage	200 V
Shutter grid frequency	25 Hz
Number of IMS averages	25
Number of points per ion mobility spectrum	500

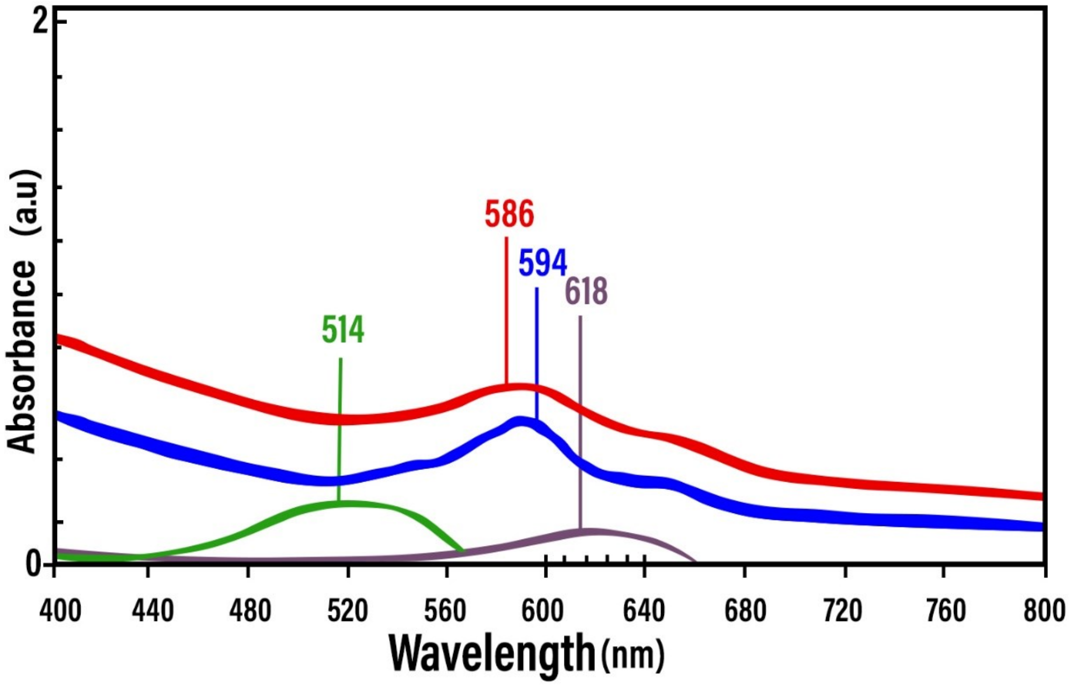

Figure S1: UV-Visible spectra of Co-MOFs based ZIF-67 prepared with different solvents, (a) methanol [594 nm], (b) ethanol [586 nm], (c) water [618 nm], (d) methanol-water [514 nm].

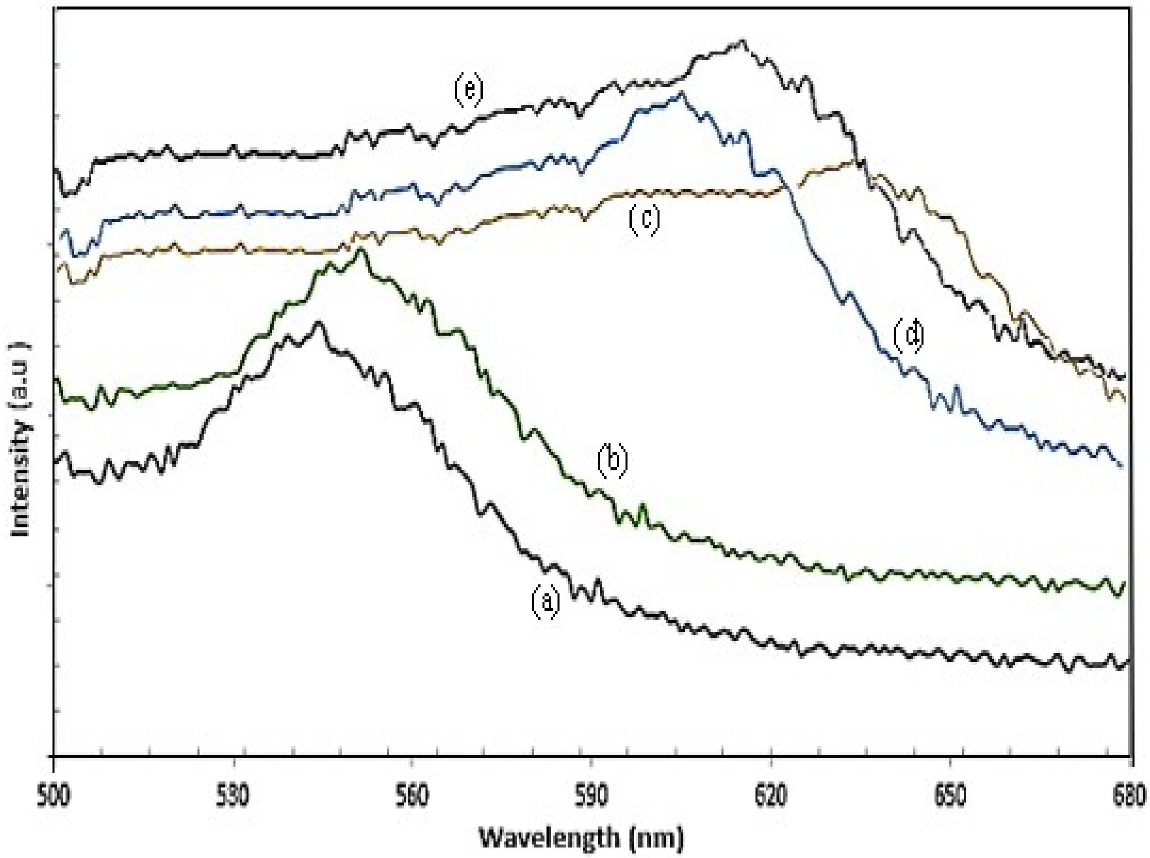

Figure S2: PL spectra of Co-MOFs based ZIF-67 Prepared with different solvents, (a) methanol-water (b) ethanol-water (c) water (d) methanol (e) ethanol.

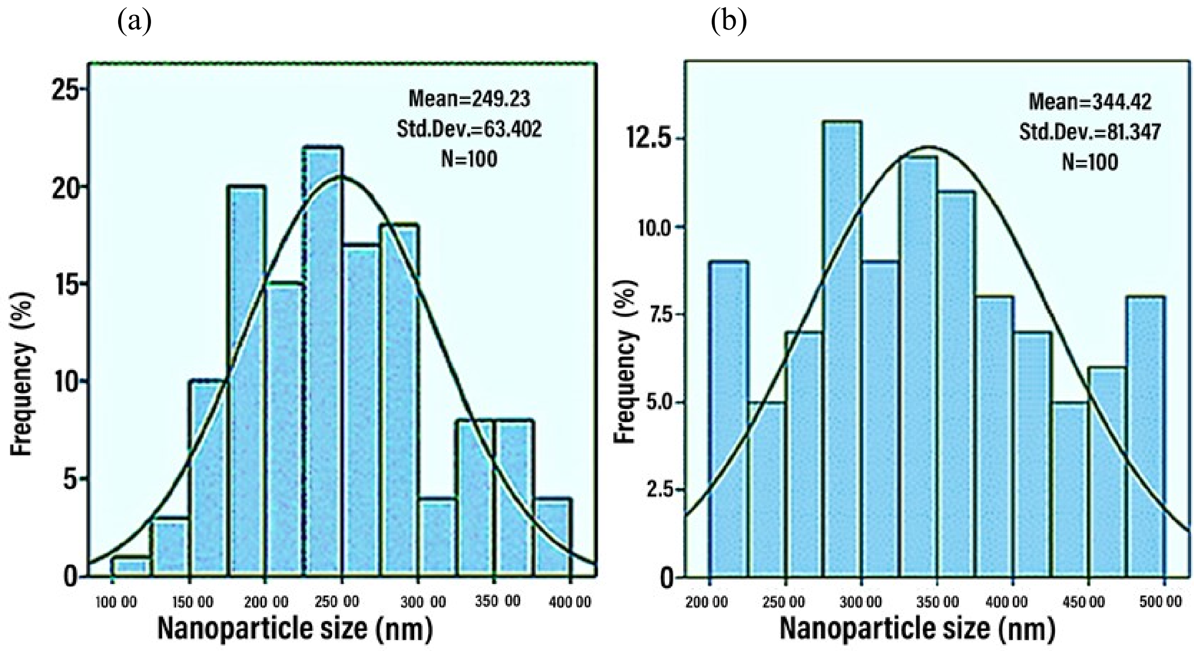

Figure S3: Particle size distribution of Co-MOFs based ZIF-67 obtained with different solvents (a) ethanol (b) methanol.

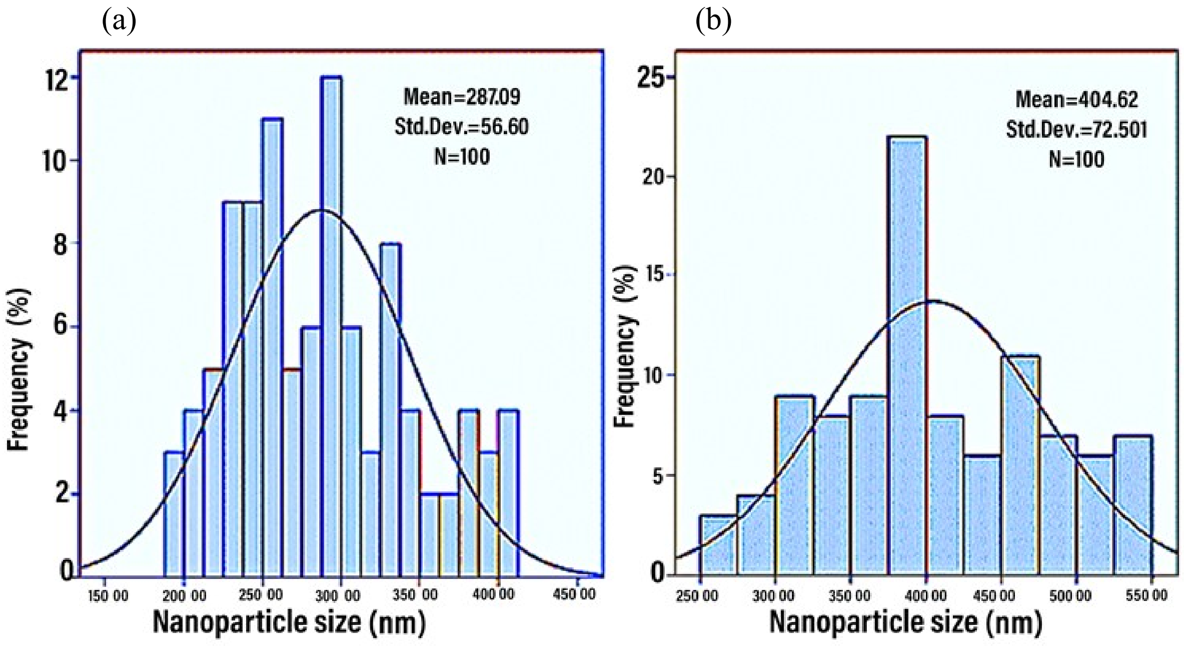

Figure S4: Particle size distribution of Co-MOFs based ZIF-67 obtained with different cobalt sources (a) $CoCl_2$ (b) $CoSO_4$.

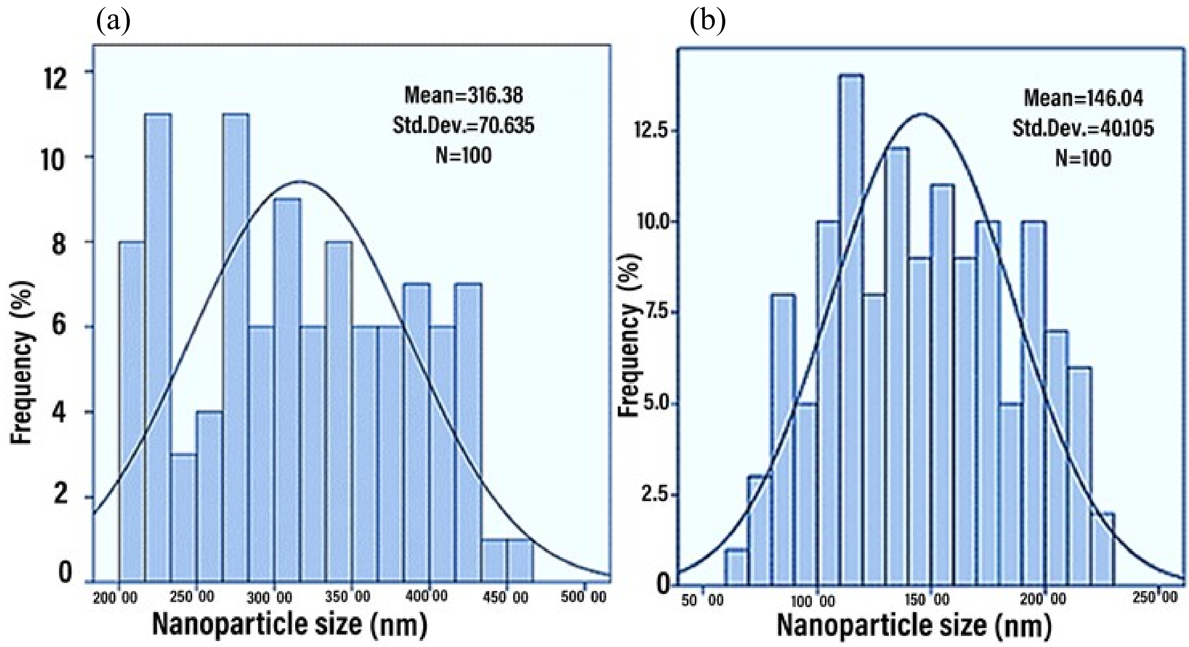

Figure S5: Particle size distribution of Co-MOFs based ZIF-67 obtained at (a) non-temperature (25°C) (b) under temperature (100°C).

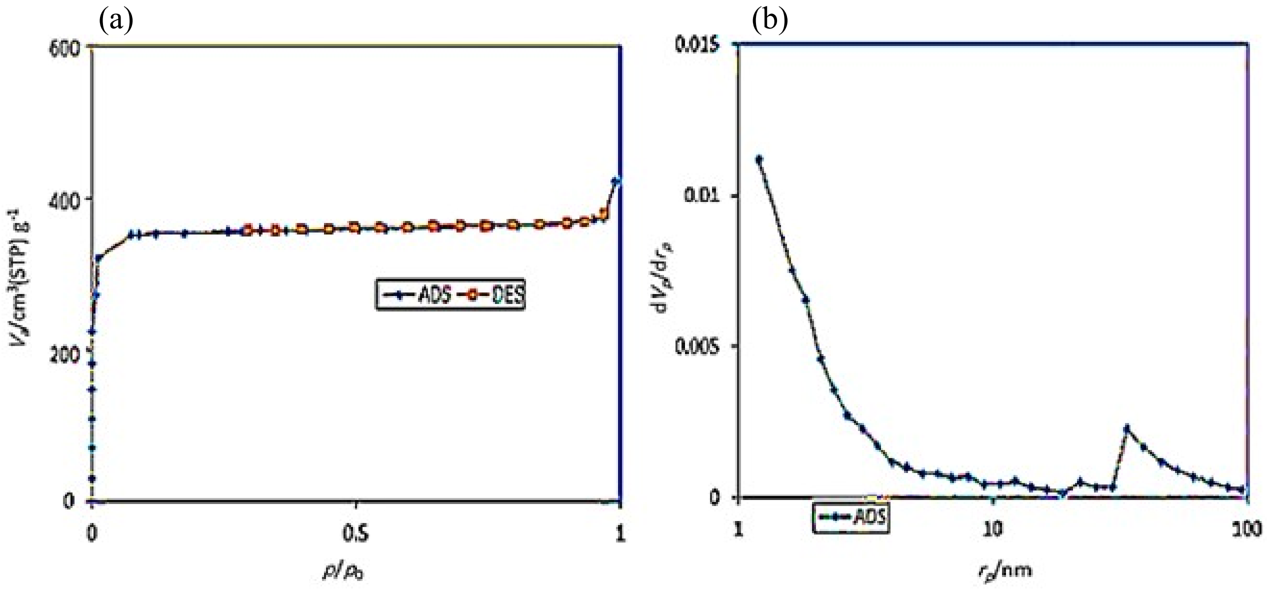

Figure S6: (a) Adsorption/desorption isotherms of N_2 and (b) pore size distribution of Co-MOFs based ZIF-67 indicate for SO_1 $sample$

Table S2: Surface area and porosity of Co-MOFs based ZIF-67 $(SO_1) sample.$

Sample	BET Surface area m^2/g	pore volume cm^3/g	Mean pore diameter (nm)
Co-MOFs based ZIF-67	1528	0.083	1.21
